# The Influence of β-Carotene and Its Liposomal Form on the Expression of EMT Markers and Androgen-Dependent Pathways in Different Prostate Cell Lines

**DOI:** 10.3390/antiox13080902

**Published:** 2024-07-25

**Authors:** Joanna Dulińska-Litewka, Kacper Dykas, Stanisław Boznański, Przemysław Hałubiec, Marta Kaczor-Kamińska, Jacek Zagajewski, Torsten Bohn, Gracjan Wątor

**Affiliations:** 1 Chair of Medical Biochemistry, Medical College, Jagiellonian University, Mikołaja Kopernika Street 7C, 31-034 Krakow, Poland; marta.b.kaczor@uj.edu.pl (M.K.-K.); jackzag@yahoo.com (J.Z.); 2 Student Scientific Group, Faculty of Medicine, Medical Bio-Chemistry, Medical College, Jagiellonian University, Mikołaja Kopernika Street 7C, 31-034 Krakow, Poland; kacper.dykas@doctoral.uj.edu.pl (K.D.); stanislaw.boznanski@student.uj.edu.pl (S.B.); przemyslaw.halubiec@doctoral.uj.edu.pl (P.H.); 3 Doctoral School of Medical and Health Sciences, Medical College, Jagiellonian University, Łazarza 16, 31-530 Krakow, Poland; 4 Luxembourg Institute of Health, Nutrition and Health Research Group, Department of Precision Health, 1 A-B, rue Thomas Edison, L-1445 Strassen, Luxembourg; torsten.bohn@gmx.ch; 5 Centre for Medical Genomics OMICRON, Medical College, Jagiellonian University, Mikołaja Kopernika Street 7C, 31-034 Krakow, Poland; gracjan.wator@uj.edu.pl

**Keywords:** prostate cancer, carotenoids, epithelial–mesenchymal transition, lipid metabolism, androgens, nuclear receptors

## Abstract

Prostate cancer (PCa) is the most common malignancy in men. Although the prognosis in the early stages is good, the treatment of advanced PCa remains a formidable challenge. Even after an initial response to hormone therapy or chemotherapy, recurrences are frequent and resistance to any systemic treatment is common. β-Carotene (BC), a plant-derived tetraterpene, is known for its antioxidant capacity and can modulate multiple cellular signaling pathways, potentially affecting androgen synthesis. We investigated the influence of BC (dissolved in EtOH/THF with a cell culture medium or encapsulated in liposomes (LP-BCs)) on the viability, migration potential, and connective tissue cleavage capabilities of several PCa cell lines (Du145, LNCaP, PC-3, and 22Rv1) and a healthy prostate model (RWPE cells). BC significantly reduced the proliferative capacity of all investigated cell lines at various concentrations (1.5–30 µM) and decreased cell migration. However, it significantly increased the expression of epidermal–mesenchymal transition (EMT) master proteins in all cancer cell lines and RWPE (*p* < 0.05) These effects were not observed with LP-BCs. This study suggests that LP-BCs, with their higher antiproliferative capabilities and pronounced inhibition of the EMT, may be a more effective form of possible PCa prevention or treatment than the free form. LPs may also modulate lipid metabolism in PCa cells.

## 1. Introduction

Prostate cancer (PCa) is the most common neoplasm in men worldwide, with around 1.4 million new cases and 375 thousand deaths per year [1]. The prostate gland is a male reproductive organ that requires androgens for normal growth and function. Prostate organogenesis is a complex process governed primarily by the action of androgens and subsequent mesenchymal–epithelial interactions [2].

Although most cases of PCa progress slowly, the disease is responsible for a significant number of deaths. PCa pathogenesis comprises the disruption of androgen-dependent intracellular signaling, resulting in an excessive cell proliferation and significant metabolism changes, such as high steroid accumulation [3], and finally, the gain independence of androgen signaling and the loss of the androgen receptor (AR). Treatment options in the late stages of the disease typically include hormone therapy and chemotherapeutics (such as docetaxel). However, they have limited effectiveness to cure the disease and are associated with adverse reactions that reduce the quality of life of patients (such as andropause symptoms after hormone therapy) [4,5]. These facts highlight the urgent need for the development of effective and safe drugs that may be used in the treatment of advanced PCa.

The nutraceutical approach is a promising direction for both prevention and therapy. Some epidemiological studies have shown that increased carotenoid intake was associated with a reduced risk of PCa development [6,7]. The exact molecular mechanism of this phenomenon is not yet fully explained. A protective effect was also observed in studies on PCa rodent models, but research regarding the mechanisms explaining these observations is still ongoing [8]. The antioxidant properties of carotenoids might be responsible for the antineoplastic effect, but other phenomena, such as changes in the expression of genes involved in survival and proliferation, have also been considered [9,10].

Cellular models can be used to investigate the molecular mechanisms of carotenoid action in PCa [11]. They can also represent the different phases of the disease, serving as a representation of the different stages of PCa tumorigenesis [12].

However, because β-carotene (BC) lacks stability and is not water-soluble, different delivery systems have been proposed. Liposomes (LPs) can be used as nanocarriers to precisely deliver drugs to the target. A variety of available surface modifications further enhances their potential as a vehicle. These particles have shown a low cytotoxicity and were successfully used in studies with other nutraceuticals [13]. In the case of PCa, only a few studies employed liposomal BC uptake and the results were not conclusive [14].

The epithelial–mesenchymal transition (EMT) is a process during which polarized epithelial cells lose tight junctions and interactions with the basal membrane and transform into mesenchymal-like cells [15]. Although the EMT is a physiological process that occurs during embryogenesis, inflammation, and wound healing, it is also a critical step in cancer development, including PCa [16]. The molecular basics of the EMT include a decrease in the expression of adhesion proteins, such as E-cadherin, zona occludens-1 (ZO-1), and occludin, but also increased levels of mesenchymal markers, such as N-cadherin, Snail, Slug, or vimentin [15]. It is also associated with the extensive breakdown of the extracellular matrix by metalloproteinases (MMPs) [17].

**Figure 1:**
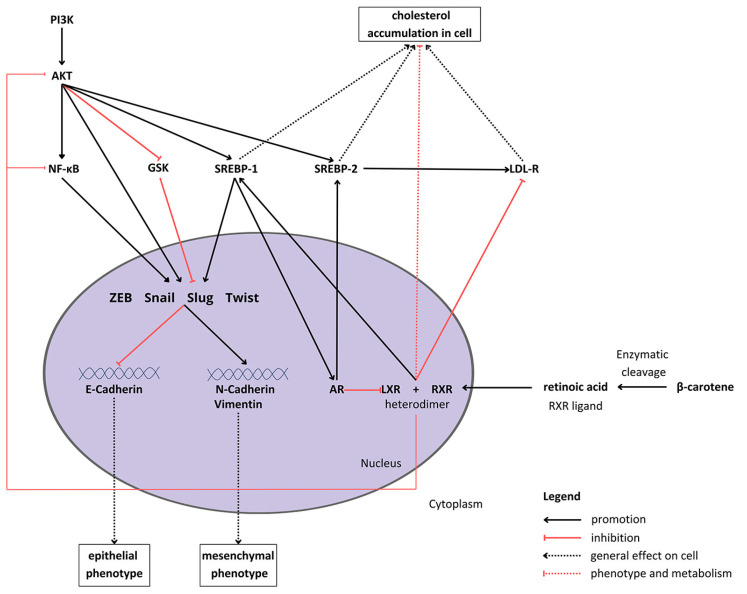
Proposed crosstalk between β-carotene, epithelial–mesenchymal transition signaling, and cholesterol metabolism in cells. Abbreviations: Akt—protein kinase B; AR—androgen receptor; GSK—glycogen synthase kinase; LDL-R—low-density lipoprotein receptor; LXL—liver X receptor, PI3K—phosphoinositide 3-kinase; RXR—retinoid X receptor; SREBP1/2—sterol regulatory element-binding protein 1/2; NF-κB—nuclear factor κB; ZEB—zinc finger E-box-binding homeobox.

The regulation of the EMT is related to intercellular signaling [18]. The potential anticancer activity of carotenoids, including BC and its metabolite (retinoic acid), implies that they might be associated with the restoration of control over the EMT (Figure 1). It was already shown in vivo in a BALB/c mouse smoking model that BC attenuates the Notch pathway, leading to the inhibition of the EMT in tobacco smoke-induced gastric cancer [19]. Moreover, the apocarotenoid, β-ionone, suppressed the EMT by downregulating the Wnt/β-catenin pathway in PCa cells [20]. Another carotenoid, lutein, seems to suppress the EMT in hypoxic conditions via the hairy and enhancer of split 1 (HES1) pathway in breast cancer cells [21].

BC metabolism consists of enzymatic conversion to retinoic acid, which binds to various receptors, such as the retinoid X receptor (RXR) [22]. The intracellular metabolic and proliferation pathways form a complex network of multidirectional connections between BC and the EMT process (what is depicted in Figure 1). For example, one of the sterol regulatory element-binding proteins (SREBPs), SREBP1, which is activated by Akt [23], raises cellular steroids levels [24]. Duanyang and colleagues showed that it also activates Snail in colorectal cancer [25]. The liver X receptor (LXR) decreases cholesterol accumulation in cells [26] by promoting its efflux and inhibiting the low-density lipoprotein receptor (LDL-R) [27]. Additionally, it suppresses Akt and nuclear factor-κB (NF-κB) signaling, responsible for proliferation and the inflammatory state, respectively [28,29]. However, LXR stimulates SREBP-1c expression [30], therefore potentially inducing the activity of the SREBP-1c/AR/SREBP-2 axis [26,31]. Carotenoids, such as lycopene, have been proven to reduce androgen-dependent AR nuclear translocation. Although they do not inhibit AR directly, they might pose antiandrogenic properties, inhibiting the expression and activity of AR [32]. However, carotenoids have not been extensively studied in the context of androgen signaling and its crosstalk with the EMT process.

Therefore, the role of carotenoids in carcinogenesis and tumor progression is still not clear [22,33].

The aim of this experimental study was to investigate how the liposomal form influences the biological effects of BC on different PCa cell lines (PC-3, Du145, and LNCaP), as well as healthy prostate cells, with a particular focus on the markers of the EMT and proteins involved in androgen signaling.

## 2. Materials and Methods

### 2.1. Cell Culture and Treatment

The research was carried out using human prostate cell lines (American Type Culture Collection, ATCC, Manassas, VA, USA) that were all derived from human PCa metastases (LNCaP, Du145, and PC-3), a PCa xenograft (22Rv1), or normal prostate cells (RWPE). The tests were performed after the 3rd or 4th passage for all types of cells. A summary of the characteristics of the used cell lines is depicted in Table 1.

The RWPE cell line was cultured in keratinocyte serum-free medium (K-SFM) enriched with bovine pituitary extract (BPE; 0.05 mg/mL) and epidermal growth factor (EGF; 5 ng/mL), and the PCa cell lines were cultured in RPMI-1640 with the addition of 10% fetal bovine serum (FBS), according to a previously described procedure [34].

Then, cells from each culture were divided into a total of eight subsets based on the added substances and exposure duration (either 24 h or 48 h): control group (without supplements), empty LP group, 3 μM BC-loaded LP (LP-BC) group, and 3 μM BC group (see Section 2.5).

Cell culturing and androgen stimulation were performed according to a previously described procedure [35]. All cell lines were tested for mycoplasma infection by polymerase chain reaction (PCR). The tests were carried out before the start of the cell culture, during, and after the completion of the research. All lines had a certificate of authentication (ATCC).

### 2.2. Preparation of LP-BCs

LPs were prepared by the hydration of the dry lipid film and following sonication. To prepare the LP-BCs, a chloroform solution of 100 mg/mL L-α-phosphatidylcholine (type XI-E from egg yolk; EYPC, 1,2-diacyl-sn-glycero-3-phosphocholine; ≥99%) was mixed with a chloroform solution of 1 mg/mL BC (weight ratio of EYPC:BC = 100:1) in a 10 mL amber glass test tube. The mixture was vortexed for 5 min. The solvent was evaporated under a gentle stream of gaseous nitrogen to complete dryness.

The dry lipid film was rehydrated with an aqueous solution of serum-free culture medium (RPMI 1640 medium with and without phenol red) and K-SFM in a volume required to achieve a final BC concentration of 25 mg/L in the loaded LPs.

Six freeze–thaw cycles with the use of liquid nitrogen and a lukewarm water bath were applied for homogenization. Finally, the samples were subjected to sonication for a total of 2 min. Titanium particles from the sonicator horn were removed by centrifugation. All samples appeared clear, and no insolubilized residues were visible.

An identical approach was used to prepare BC-free LPs, except for the step of adding a BC solution to a solution of L-α-phosphatidylcholine.

**Figure 2:**
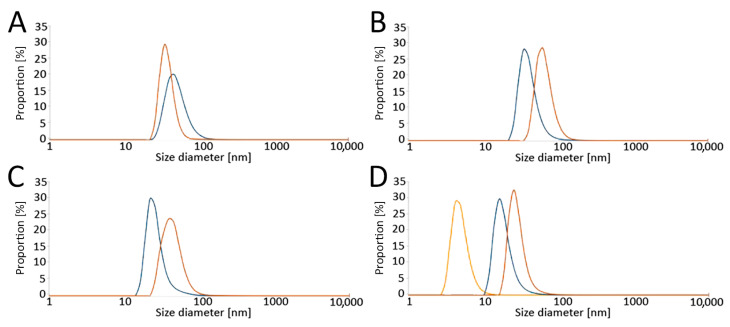
Analysis of liposome sizes by dynamic light scattering (DLS) in (**A**) RPMI without phenol red, (**B**) RPMI with phenol red, and (**C**) K-SFM, as well as (**D**) the effect of serum on liposome diameter (ratio of serum: RPMI with phenol red medium = 1:1). Size distributions of the liposomes are presented. Color legend: blue—empty liposomes, orange—carotene-loaded liposomes, yellow—serum. Abbreviations: RPMI: Roswell Park Memorial Institute medium; K-SFM: Keratinocyte-SFM Basal medium.

The size distribution of the obtained LPs was determined by a Zetasizer Nano ZS Instrument (A.P. Instruments, Warsaw, Poland) using dynamic light scattering (DLS). Samples were measured at 25 °C with 173° scattering. The results are presented as number-based distributions (Figure 2).

### 2.3. Analysis of Purity and Stability of BC

The stability and concentrations of BC preparations in culture media were measured through the original method described by Wertz et al. [34] and modified by Dulińska et al. [35].

All reagents—acetone, acetonitrile (ACN), tert-butyl methyl ether (tBME), ethanol (EtOH), tetrahydrofuran (THF) and 2,6-di-tert-butyl-p-cresol (BHT), ammonium acetate, and triethylamine—used for the high-performance liquid chromatography (HPLC) anaysis were of analytical grade (Sigma Aldrich, Darmstadt, Germany). Acetone, ethanol, and tetrahydrofuran solutions were always filtered through an aluminum oxide column before use.

### 2.4. Cytotoxicity Assay

All cell lines were seeded in triplicate into 96-well culture plates (FALCON, 4.5 × 10^3^ cells/well) in 100 µL of a culture medium and left in the incubator for 24 h to adhere to the well surface.

After this time, the culture medium was replaced with 100 µL of a new one containing the control medium, LPs, BC, or LP-BCs. Each cell line was incubated for 24 h and 48 h under culture conditions.

Then, 5 μL of 3-(4,5-dimethylthiazol-2-yl)-2,5-diphenyltetrazolium bromide (MTT) solution (5 mg/mL in phosphate-buffered saline (PBS)) was added to each well, and the plates were further incubated for 3 h under culture conditions.

After this, 50 μL of lysis buffer (10% sodium dodecyl sulphate (SDS) in 0.01 M HCl) was added, and the plates were further incubated overnight. The absorbance in each well was measured at 570 nm using an enzyme-linked immunosorbent assay (ELISA) on a plate reader (Synergy HT, Multi-Detection Microplate Reader, BIO-TEK^®^INSTRUMENTS, INC., Winooski, VT, USA), and the data were analyzed using the KC4 Software, version 3.4 (BIO-TEK, Charlotte, VT, USA).

In order to confirm the effect of BC on cells, cytotoxicity assays using the LDH method were also used. Cells were seeded in triplicate into 96-microwell plates (FALCON) at a density of 2–4 × 10^3^ cells per well and incubated without or with BC (1.5 μM, 3.0 μM, 5 μM, 7.5 μM, 15.0 μM, and 30.0 μM) for 24, 48, and 72 h. Afterwards, culture growth media were mixed with the reaction mixture from the Cytotoxicity Detection Kit (Roche Diagnostics GmbH, Mannheim, Germany. The reaction was stopped with 1 M HCl. The colorimetric assay for the quantification of cell death was based on the assay of the activity of lactate dehydrogenase (LDH) released from the damaged cells into the supernatant. The absorbance of the colored product—formazan—was measured at 490 nm by a plate reader (Synergy HT, Multi-Detection Microplate Reader, BIO-TEK^®^INSTRUMENTS, INC., Winooski, VT, USA, Microplate Date Analysis Software KC4).

The percentage cytotoxicity was determined according to the percentage cytotoxicity formula provided in the manufacturer’s protocol.

### 2.5. Preparation of Cell Culture Media Supplemented with BC

To prepare appropriate solutions, the crystalline BC standard was initially dissolved in an EtOH/THF solution (1:1 ratio) to obtain a stock solution with a concentration of 3 mM BC, which was then diluted to the appropriate concentrations of 1.5, 3.0, 7.5, 15.0, and 30.0 µM in the cell culture medium.

To minimize BC degradation, all procedures were performed in the dark with small amounts of BC placed in amber vials overlayed with argon.

All cell cultures with BC were carried out in a specially prepared room with the lighting turned off and the windows secured (tinted foil) in order to minimize the impact of light.

### 2.6. Cellular Uptake of BC Measured with HPLC

The BC preparations were added to different prostate cell lines (LNCaP, PC-3, Du145, and RWPE) as described above. After 24 h and 48 h, the cells were washed 3 times with the PBS solution. To separate the cells from the cultured plates, they were washed again with PBS, detached from plates by trypsinization, pelleted (centrifuged for 3 min at 8000× *g*), and stored frozen (at −80 °C) until further analysis.

The cellular uptake of BC by the prostate cell lines was measured using a Shimadzu 10 HPLC system (Kyoto, Japan) series, consisting of LC10At vp pump, Dgu-14 A degasser, CTO-10Asvp column oven, Sil-10ADvp autosampler, and an SPDM10Avp diode array detector.

BC from the media and cell extracts was separated using a Vydac 218TP54 C18 column (4.6 × 250 mm, 5 µm 300 A) with a guard column with similar packing. The mobile phase consisted of ACN/tBME/80 mM ammonium acetate/triethylamine—73:20:7:0.5 *v*/*v*. The BC separation process was carried out in an isocratic system with a constant flow of 1.5 mL/min, and detection was carried out using a diode array detector at 450 nm [36].

**Figure 3:**
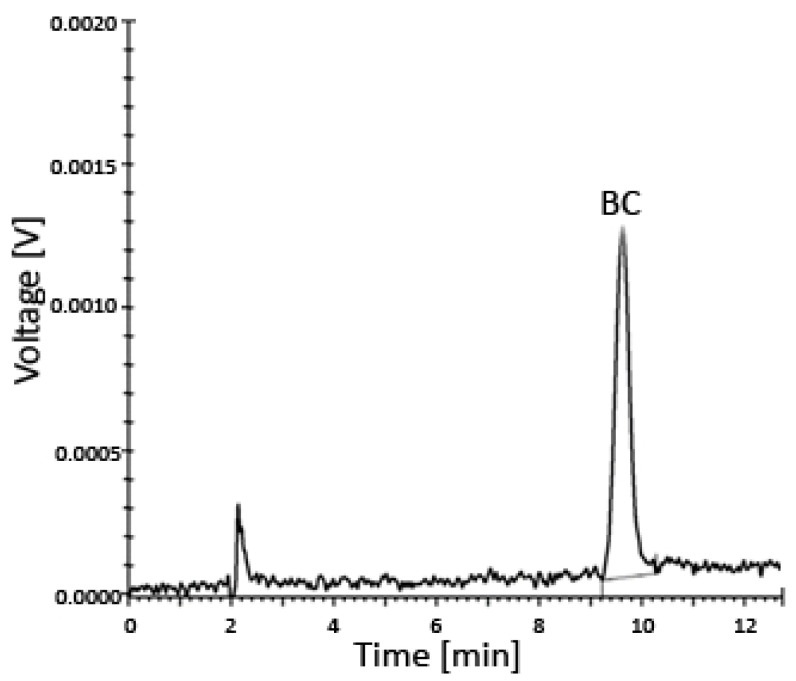
Uptake of BC by prostate cells incubated for 24–48 h in various media with different concentrations of BC analyzed using HPLC—a representative chromatogram. The limit of detection (LOD) was 0.024 µM and the limit of quantitation (LOQ) was 0.049 µM. Abbreviations: BC—β-carotene; HPLC—high-performance liquid chromatography.

**Figure 4:**
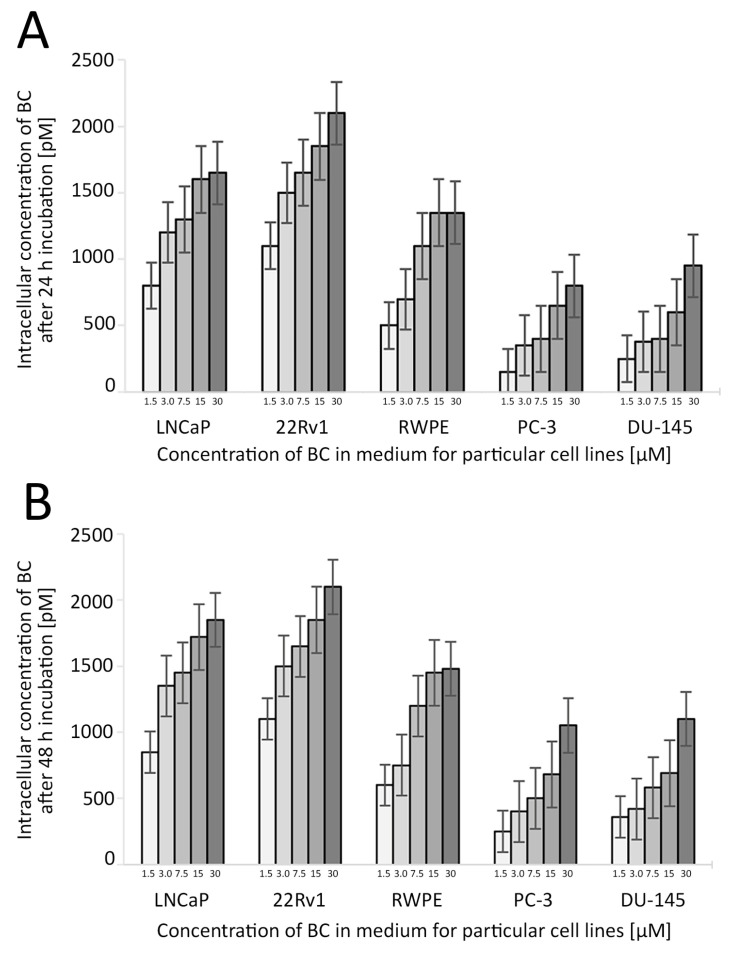
The uptake of BC in the studied cell lines depending on the BC concentration and time of the treatment. The concentration of BC used in further experiments was 3 µM. Whiskers represent the standard error of n = 3 replicates. Intracellular concentration of BC after 24 h (**A**) and 48 h (**B**) incubation (pM). Abbreviations: BC—β-carotene.

The entire system was under the control of the controller unit CBM-20A, while all data were collected and processed using the Shimadzu Lab solution software (Roche, column–C18, 218TP54, Vydac, Shimadzu SCL-10AVP instrument (Shimadzu, Kyoto, Japan) with the SPD-10AV detector) (Figure 3 and Figure 4).

BC extraction from the cell pellets was carried out as previously described [36,37].

### 2.7. Proliferation Assay

The proliferation of cells was assessed with the crystal violet assay as previously described [38].

Briefly, cells were seeded in triplicate in 96-microwell plates at a density of 2–4 × 10^3^ cells/well (depending on the cell line) and cultured in the control medium or with BC for 24 h or 48 h. The cells were exposed to BC at different concentrations (1.5 μM, 3.0 μM, 7.5 μM, 15.0 μM, and 30.0 μM).

Furthermore, colorimetric immunoassay ELISA BrdU (Roche) tests were performed as described previously [37]. The absorbance was measured at 450 nm on an ELISA plate reader. The selected concentration of 3.0 μM of BC was studied with and without LPs for the 24 h and 48 h treatments, as previously described [37,38].

### 2.8. Wound Healing Assay and Migration Assay

Cells were grown until confluence in 24-well plates for the wound healing assay. A small linear scratch was made in the confluent monolayer by a gentle move with a sterile pipette tip.

Cells were washed with a medium to remove cellular debris before treatment with serum-free OptiMEM media with supplements, i.e., BC, LP-BCs, LPs, or EtOH/THF as a control.

Twenty-four hours later, images of the migrated cells were taken using a digital camera (Nikon, Tokyo, Japan) connected to the inverted microscope. The analyses were repeated three times in duplicate.

### 2.9. RNA Extraction and Reverse Transcription

The harvested cells were immediately transferred to the freezer and stored at −80 °C. Total RNA was isolated using the Maxwell RSC RNA simply RNA Cells Kit (Promega, Madison, WI, USA). The procedure applied paramagnetic particles (as a mobile solid phase) and the Maxwell RSC 48 Instrument (Promega, Madison, WI, USA). The concentration and purity of RNA were determined with the use of Nanodrop (Thermo Fisher Scientific, Waltham, MA, USA). Next, the RNA (0.1–1.0 μg) was reverse-transcribed to cDNA using MultiScribe Reverse Transcriptase, according to the manufacturer’s protocol (High-Capacity cDNA Reverse Transcriptase Kit, Thermo Fisher Scientific, Waltham, MA, USA).

### 2.10. qPCR

For the gene expression analysis, we applied quantitative PCR with the hydrolysis probe method. We used a validated TaqMan assay (Thermo Fisher Scientific). Overall, we analyzed 5 transcripts for genes: AR (id: Hs00171172_m1), TNF (id: Hs00174128_m1), HMGCR (id: Hs00168352_m1), PIK3C3 (id: Hs00176908_m1), and SREPB2 (id: Hs01081784_m1). As an endogenous control, we used 18S ribosomal RNA (id: Hs03928985_g1). Briefly, 10-fold diluted cDNA and a specific assay were added to the TaqMan gene expression Maser Mix (Thermo Fisher Scientific). The real-time reaction was carried out on the CFX384 thermal cycler (BioRad, Hercules, CA, USA) for 40 cycles, according to the manufacturer’s protocol. All samples were processed in triplicate with the endogenous control at the same 384 format plate. After the end of the reaction, Cq data (cycle threshold set up at 500 RFU) were transferred to the R software for analysis (version 4.3.2).

### 2.11. Gene Expression Analysis

Gene expression was measured as the relative expression using the ΔΔCt method with 18S rRNA as an internal control (reference gene) and 24 h/48 h cell culture as a reference group [39]. The main calculations were performed using the “pcr” package of R [40] with the control group (24 h cell culture) set as 1 and default mode “separate tube”. The Wilcoxon test was used to compare the reads at 24 h and 48 h for each transcript. The data visualization was made in R using the “ggplot2” version 3.4.4 and “pheatmap” packages version 1.0.12.

### 2.12. Western Blot Analysis

Western blot analysis (cells lysis, Western blot, detection, and visualization of blots) was carried out as previously described [34,38]. The list of primary antibodies used to detect the investigated proteins is presented in Table 2. A Qubit^®^ Protein Assay Kit (Thermo Fisher Scientific, Pierce Biotechnology, Rockford, IL, USA) was used to determine the protein concentration of each sample. Cell lysates containing equal amounts of proteins (30 μg) were separated on 10% SDS-PAGE gels and transferred onto the membranes (PVDF Western Blotting Membranes—Merck Life Science Sp.z.o.o., an affiliate of Merck KGaA, Darmstadt, Germany).

The cells were filtered with Millipore and incubated with horseradish peroxidase (HRP)-conjugated goat anti-rabbit (Sigma-Aldrich, Darmstadt, Germany) or horse anti-mouse (Sigma-Aldrich, Darmstadt, Germany) IgG secondary antibodies (dilution 1:3000). Protein immunoreactivity was detected by chemiluminescence, and images were recorded with a ChemiDocTM MP Imaging System (Bio-Rad, Hercules, CA, USA). Molecular masses were estimated through reference to standard proteins (Thermo Fisher Scientific, Pierce Biotechnology, Rockford, IL, USA). The densitometric analysis of proteins was performed using the Image Lab Software, version 6.0 (Bio-Rad, Hercules, CA, USA). Representative membranes from at least two or three independent experiments are shown.

### 2.13. Statistical Analysis

All calculations were made in the R environment with the “pcr” and “pheatmap” packages. As a default, we used the ΔΔCt method with 18S as the reference gene and the 24 h control experiment as the reference group [39].

Comparisons between continuous variables were performed by ANOVA. Dunnett’s post hoc comparison test determined which values differed significantly from the controls. Tukey’s multiple comparison test was used for data in which the differences between individual groups were studied. All analyses were conducted using the Statistica 13 software (StatSoft Inc., Tulsa, OK, USA). The probability of type I error (2-sided) was presented as * *p* < 0.05, ** *p* < 0.01, and *** *p* < 0.001.

## 3. Results

### 3.1. Cytotoxicity

No cytotoxic effect was noted for the further evaluated concentrations of BC, LPs, and LP-BCs (cell viability > 85% in all samples; Table 3).

### 3.2. Proliferation of Cells

BC in concentrations ranging from 1.5 µM to 7.5 µM were associated with a significant reduction in the proliferative capacity of all investigated cell lines compared to the controls. For LNCaP and PC-3, a clear U-shaped relationship between the BC concentration and the proliferation rate was observed, with the peak of growth inhibition at 3–5 µM of BC. Interestingly, at 15 µM of BC, these cell lines exhibited a higher proliferation than the control samples. For the cell line 22Rv1, such effects were observed after 24 h but not 48 h. Exposure to 30 µM BC significantly decreased the proliferation of all cell lines (in 22Rv1, such a response was only visible after 48 h of culture).

**Figure 5:**
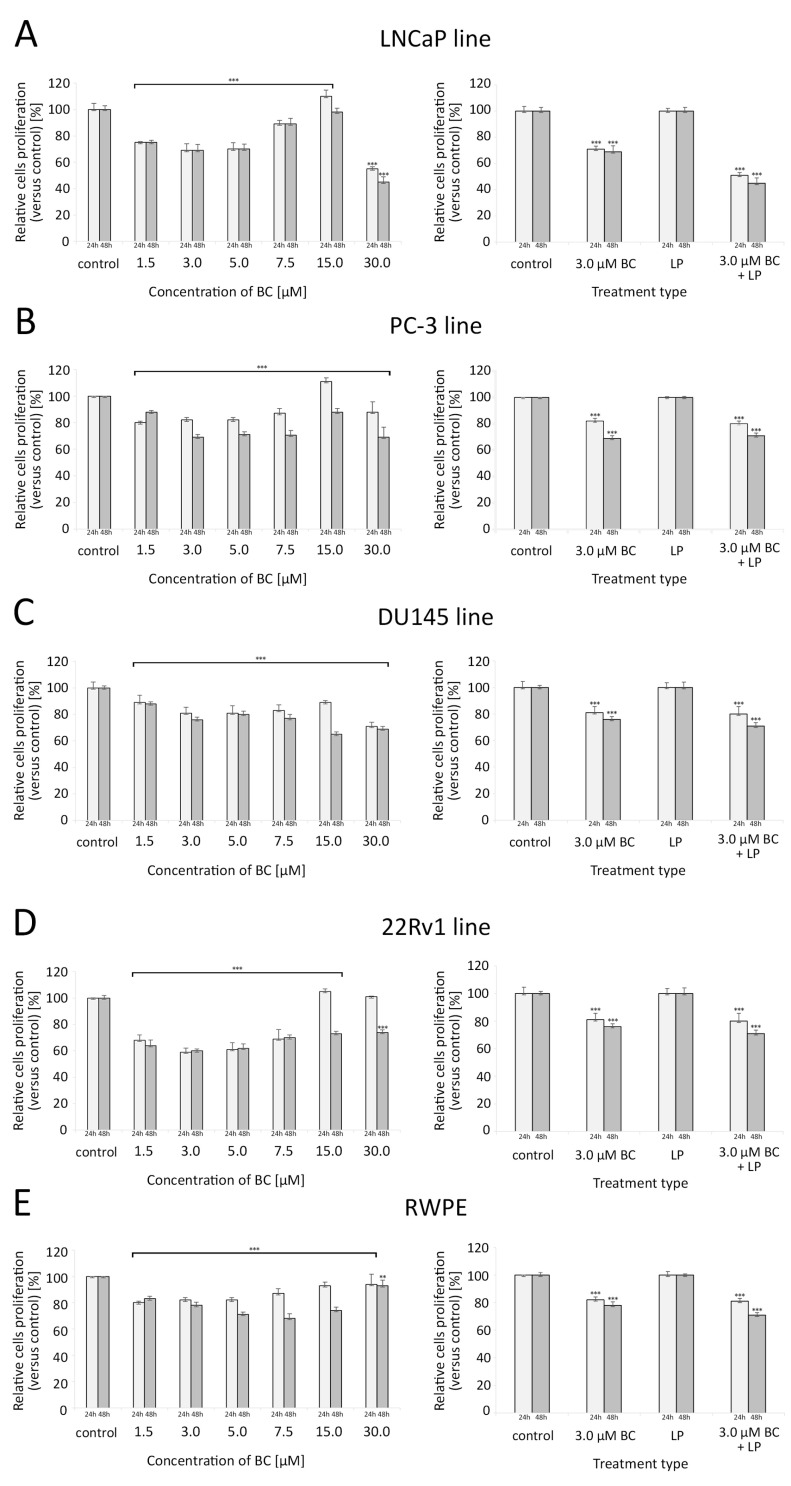
Proliferation of cells assessed with the crystal violet assay (for different concentrations of BC—5 series in triplicate) and with the ELISA BrdU colorimetric immunoassay (for LPs and/or BC at 3.0 µM—4 series in triplicate) after 24 h and 48 h exposure. Comparisons were made with unpaired *t*-tests to the respective control culture. (**A**) LNCaP line, (**B**) PC-3 line, (**C**) DU145 line, (**D**) 22Rv1 line, (**E**) RWPE line. * *p* < 0.05, ** *p* < 0.01, and *** *p* < 0.001. Abbreviations: BC—β-carotene; LPs—liposomes.

In all cell lines, exposure to 3 µM BC (with or without LP) caused a significant reduction in the proliferation rate, and this effect was more pronounced after a longer incubation time. LP-BCs were associated with a stronger decrease in proliferation levels than BC alone in the LNCaP and 22Rv1 cell lines (*p* < 0.001). For further details, please see Figure 5.

### 3.3. Wound Healing/Migration Assay and Zymography

**Figure 6:**
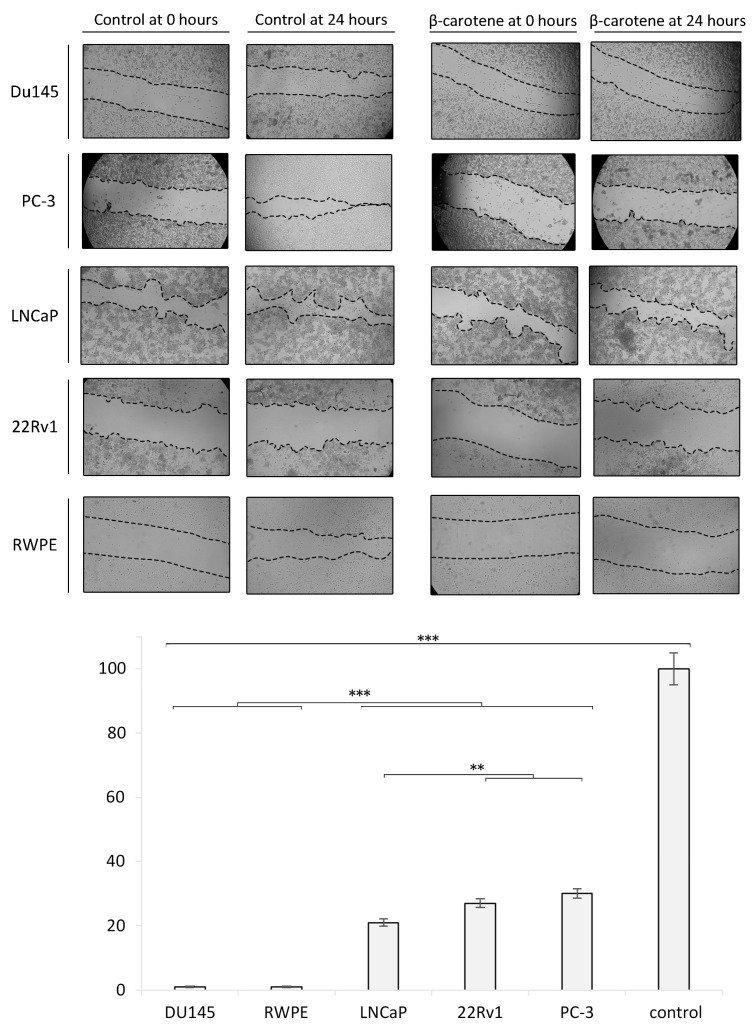
Wound healing/migration assay in vitro. The confluent cell monolayer of each investigated cell line was wounded after 24 h of growth, and then, repeated photographs were taken with a digital camera connected to an inverted microscope. The wound closure area was measured with ImageJ and is presented as the ratio of the closed wound (versus the control sample). Diagram presents the data from the wound healing/migration assay in a quantitative representation of data (mean ± SD) from three independent experiments. Values are denoted as * *p* < 0.05, ** *p* < 0.01, and *** *p* < 0.001.

Cells exposed to 3 µM of BC for 24 h showed a markedly reduced wound healing. An almost complete inhibition of migration was observed in the Du145 and RPWE cell lines. LPs, BC, and LP-BCs increased the activity of MMP-7 in LNCaP cells. MMP-2 activity was particularly high in LNCaP cells treated with BC for 24 h and with LPs for 48 h. For further details, please see Figure 6.

### 3.4. Western Blot Analysis

**Figure 7:**
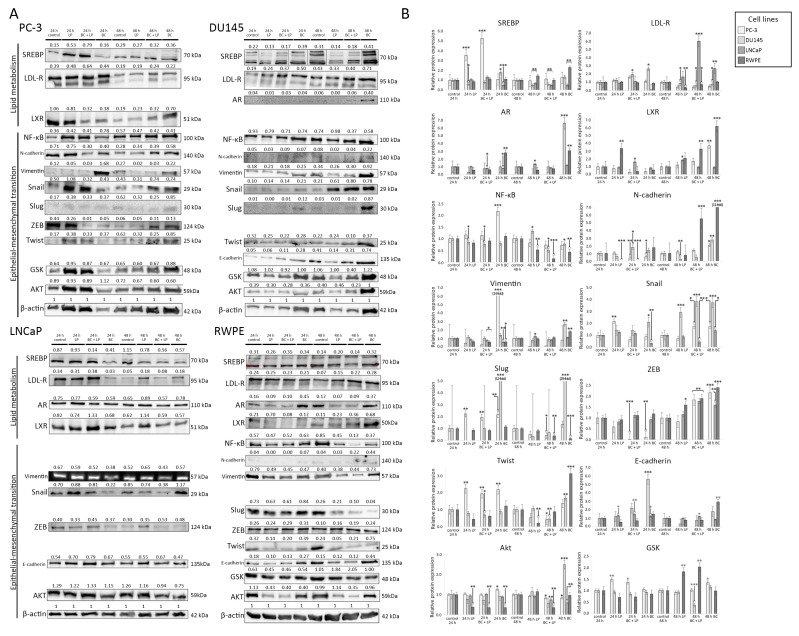
(**A**) The results of the Western blot analysis. The relative protein expression compared to β-actin (loading control) is presented. The order of reads for SREB in Du145 was rearranged to preserve consistency with the order of samples in the remaining tests. (**B**) The relative abundance of different proteins measured with Western blot (in comparison to the control sample). The histograms *p*-values (versus control) are denoted as * *p* < 0.05, ** *p* < 0.01, and *** *p* < 0.001. The quantity of proteins is presented in comparison to their amount in the control sample (after the same time of observation). β-actin was used as a protein loading control. Abbreviations: Akt—protein kinase B; AR—androgen receptor; GSK—glycogen synthase kinase; LDL-R—low-density lipoprotein receptor; LXL—liver X receptor, PI3K—phosphoinositide 3-kinase; RXR—retinoid X receptor; SREBP—sterol regulatory element-binding protein; NF-κB—nuclear factor κB; ZEB—zinc finger E-box-binding homeobox.

**Figure 8:**
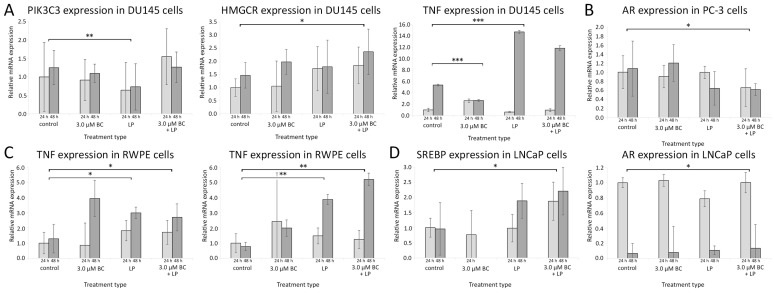
The relative gene expression levels measured with the RT-qPCR method with reference to the control samples after 24 h and 48 h. The histogram values are a quantitative representation of data (mean ± SD) from three independent experiments. (**A**) DU145 line, (**B**) PC-3 line, (**C**) RWPE line, (**D**) LNCaP line. Statistically significant differences are denoted with asterisks (* for *p* < 0.05 ** for *p* < 0.01, and *** *p* < 0.001). Only results with at least one statistically significant difference between samples are presented for the clarity of data presentation. Abbreviations: AR—androgen receptor; HMGCR—3-hydroxy-3-methyl-glutaryl-coenzyme A reductase; PI3K—phosphoinositide 3-kinase; SREBP—sterol regulatory element-binding protein; TNF-α—tumor necrosis factor-α.

The photographs of Western blot membranes with the relative abundance of proteins (corresponding to the amount of β-actin) are presented in Figure 7. Plots presenting the relative expression of the proteins are shown in Figure 8. We considered a change in protein amount as a “decrease” if it was less than 0.5 times and as an “increase” if it was more than 2.0 times of the control sample (for a given time of observation).

#### 3.4.1. Incubation with Empty LPs

In the PC-3 line, 24 h incubation with empty LPs induced the overexpression of SREB, Snail, Slug, and Twist, while vimentin was downregulated. Exposition for 48 h to the empty carrier downregulated LDL-R and vimentin.

The incubation of Du145 with LPs for 24 h downregulated the expression of AR. Slug was undetectable after 24 h. After 48 h exposure, AR was undetectable. Snail was overexpressed, while E-cadherin and N-cadherin showed a lowered expression than the control.

The empty LPs did not affect the expression of proteins in the LNCaP cell line.

The RWPE line exhibited a decrease in Akt and Twist expression for both 24 h and 48 h. N-cadherin was undetectable after 24 h and 48 h. After 48 h, LDL-R was upregulated.

#### 3.4.2. Incubation with LP-BCs

PC-3 cells treated with LP-BCs for 24 h showed a decrease in LXR, N-cadherin, and vimentin expression (compared to the control), while SREB was upregulated. After 48 h, vimentin, Slug, and Twist were lower than in the control group.

In the Du145 cell line, after 24 h, E-cadherin was increased compared to the control. Simultaneously, after the 48 h treatment with LP-BCs, N-cadherin, GSK, NF-kβ, and Twist were lower than in the control, and Snail was higher than in the control.

LNCaP exposed to LP-BCs for 48 h showed downregulated Snail and SREB but upregulated LDL-R.

After 24 h incubation with LP-BCs, RWPE cells had decreased Akt and LXR. Moreover, N-cadherin was undetectable. After 48 h, a downregulation of Akt, NF-kβ, and Slug was observed, while GSK, LDL-R, and LXR were increased.

#### 3.4.3. Incubation with BC

After 24 h of treatment with BC, the PC-3 line exhibited a downregulation of LXR and ZEB, while NF-kβ, Slug, Twist, and vimentin were overexpressed. Exposure for 48 h to BC increased the expression of LXR, N-cadherin, and ZEB.

Du145 cells showed an increased expression of E-cadherin, LDL-R, Snail, and Slug after 24 h. After 48 h, AR was re-expressed in the BC group. Additionally, higher Akt, N-cadherin, Snail, Slug, and vimentin levels were observed.

After the BC treatment for 24 h, the LNCaP line was characterized by a decrease in LDL-R, Snail, and SREB. A longer 48 h exposure decreased SREB but increased LDL-R.

The RWPE line exhibited an increased AR and decreased Akt after exposure to BC for 24 h. After 48 h, the levels of AR, E-cadherin, LXR, N-cadherin, Twist, SREB, LDL-R, and ZEB were increased, but NF-kβ and Slug were decreased.

### 3.5. RT-qPCR

The data from RT-qPCR are presented in Figure 8 and Figure S1 from Supplementary Materials.

#### 3.5.1. Influence of Incubation with Empty LPs

After treating Du145 cells with the empty LPs, the PIK3C3 mRNA expression was significantly lower than in the controls (*p* < 0.01). In RWPE cells, the level of mRNA for HMCGR and TNF-α was significantly higher compared to the controls (*p* < 0.01 and <0.05, respectively). No statistically significant change in gene expression patterns was observed for LNCaP and PC-3 cells.

#### 3.5.2. Incubation with LP-BCs

PC-3 cells exposed to LP-BCs showed downregulated mRNA for AR when compared to the control (*p* < 0.05). In LNCaP cells, the expression of SREBP mRNA was increased (*p* < 0.05). Higher levels of HMGCR mRNA were observed in Du145 and RWPE cells, while the TNF-α mRNA expression was elevated only in RWPE cells.

#### 3.5.3. Incubation with BC

Exposure to sole BC was associated with elevated mRNA levels for AR in the LNCaP cell line and reduced TNF in Du145 cells (only after 48 h).

## 4. Discussion

Our study demonstrated that, in the presence of BC at 3 µM and LP-BCs, both healthy prostate and PCa cell lines respond with numerous changes in the level of intracellular signaling. In particular, this involved pathways associated with lipid metabolism and with the regulation of EMT. Our findings indicate that the LP treatment alone does not affect cell proliferation. However, LPs were shown to modulate intracellular signaling related to steroid metabolism and the EMT. LP-BCs, however, also decreased cell proliferation and migration. The analyses of protein expression showed a downregulation of mesenchymal markers, followed by a rise in the expression of E-cadherin and proteins responsible for higher steroid efflux.

BC is a common component of the human diet, abundant in plant-derived foods, particularly in orange and red vegetables [41]. An important basic molecular mechanism of action depends on the conversion into retinol (vitamin A) by β-carotene oxygenases, such as BCO1 and BCO2 [41], and which might then be further processed into all-trans-retinoic acid [42,43]. Moreover, BC shows antioxidant properties resulting from the presence of numerous conjugated double bonds [44]. In this regard, carotenoids and their metabolites may interact with cellular pathways via transcription factors, including NF-kB and Nrf2 [45].

Its concentration in human serum after supplementation might be higher than 5 µM, as was shown in several studies [46,47], although generally, i.e., based on a normal diet, it is not higher than 1 µM [48].

In previous studies, the growth inhibitory effects of BC on Du145, LNCaP, and PC-3 cell lines were observed only at higher concentrations (>10 µM), which are arguably hard to achieve in vivo [49,50]. Despite these observations, the data from prospective studies with human subjects suggested that the supplementation of BC and maintaining its high serum concentration might reduce the risk of PCa development [51,52]. The exact mechanisms explaining the observed discrepancies remain elusive; however, some data suggest that the way of response to oxidative stress might play a significant role here (e.g., the polymorphisms of superoxide dismutase might influence the effects of BC against PCa) [52].

The exact minimal efficient concentration of BC is difficult to establish. As our results showed, in the case of the LNCaP and PC3 lines, a U-shaped relationship between the BC concentration and proliferation was found with the control group (0 µM) and higher (>15 µM) concentrations seemed to be less effective than 3 and 5 µM. A similar profile was achieved for the 22Rv1 line, especially for a shorter incubation period. Katake-Nara et al. [53] also found that other carotenoids, like capsanthin, might suppress cellular functions in lower concentrations more efficiently than in higher ones. However, there are few studies investigating the effect of BC at low concentrations; so, the U-shaped relationship could be omitted and not detected in similar studies. Low concentrations were tested for lycopene, but no U-shaped results were achieved [54]; rather, a dose-dependent suppression of viability was confirmed. On the other hand, there is a study [55] exhibiting a profile similar to our results for the LNCaP and PC3 lines for lycopene, especially after a longer treatment period (72 h), where the concentrations of 0.5, 1.0, and 5.0 µM were more effective in viability reduction than both extremely low (0.1 µM) and high (10.0 µM) concentrations.

It is worth mentioning that, in biological systems, the over-excessive supplementation of some substances might have a harmful effect, but in the described case, concentrations that have an adverse effect (>15.0 µM of BC) are difficult to achieve. The U-shaped effect of BC might be related to cell-cycle arrest, which was found to be triggered by BC and lycopene [56]. It is possible that higher concentrations might overcome this arrest-triggering proliferation. We also need to consider the fact that, in cellular models, we cannot mimic the effects of BC on the immune system, as was proven for lycopene [57]. Further studies are needed to confirm the observed relationship and to explain its mechanism.

The migratory effect was confirmed to be decreased in our study, as is summarized in Figure 6. Similar results were observed for other carotenoids, such as lycopene [58], which was able to suppress migration in PC3 and Du145 cells. It is worth mentioning that, in both our and the cited study, low concentrations of carotenoids, which can be easily achieved in human plasma, have an ability to reduce the migratory potential.

Studies showed that LP-BCs (at 33.3 mg/kg BC) can reduce tumor growth in animal models [59]. Unfortunately, the impact of LP-BCs on PCa is only poorly understood. LPs alone were proven to be efficient drug carriers, reducing toxicity and increasing both efficiency and the therapeutic index [60,61]. In contrast to the BC treatment, LP-BCs inhibited signaling pathways related to EMT progression and steroid accumulation. It is possible that LP delivery systems reduced the BC dosage needed for the mentioned effect. The hardly noticeable impact of BC alone on gene expression suggests its low stability, which is enhanced by LPs. This also contributes to a higher efficiency in the intracellular activity of BC at a low dosage. It corresponds with a study conducted by Williams et al., who pointed out the importance of BC carriers [14]. On the other hand, there is some evidence that LPs might not be the best carriers of BC as they do not increase its uptake compared to other methods in PC-3 and Du145 cells [14].

Previous research [37] showed that low doses of BC induce c-Myc activity, which participates in EMT signaling [62]. At the concentration of 3 µM, solely BC treatment induced bidirectional changes, simultaneously inhibiting and promoting EMT-related cellular pathways. Further analyses showed that 48 h BC exposure stimulated EMT progression. Furthermore, the low dosage of BC induced the expression of N-cadherin, physiologically absent in the prostate epithelium in the healthy RWPE cell line and caused the re-expression of AR in Du145.

Only few studies investigated the impact of BC on EMT intracellular signaling. Fang and colleagues found that β-ionone, a BC terminal analog, inhibits Wnt/β-Catenin signaling at the concentration of 60–180 µm in PC-3 and 22RV cells, resulting in decreased N-cadherin levels [20]. Additionally, the authors suggested that the β-ionone effect is caused by its binding to RXR. Despite the fact that a different compound at a higher concentration was used, it may lead to the conclusion that a higher concentration of active BC, achieved by the usage of LPs, can also induce similar effects.

However, other models of PC, for example, a murine tumor xenograft derived from the PC-3 line, seem to confirm observational studies in humans. As Yang et al. showed [63], BC supplementation (16 mg/kg twice a week for 7 weeks) results in a reduction in the tumor xenograft volume. Surprisingly, BC was confirmed to increase VEGF in plasma, which generally enhances the angiogenic potential of cells, but the overall effects seemed to be beneficial. It has been shown that BC intake (10 mg/kg body weight/day) reduces Notch pathway activity in tobacco-induced gastric cancer in mice, although the molecular mechanism of this outcome remains unknown [19]. In the PC-3 line in our study, LP-BCs downregulated another crucial protein for the progression of neoplasms, i.e., N-cadherin, which confirms its favorable effect.

It was previously shown that BC at very high concentrations (>30 µM) slows the proliferation of the PC-3, Du145, and LNCaP cell lines [64]. Similarly to our results, even high concentrations of BC did not exhibit cytotoxicity. Palozza et al. [65] indicated that only PC-3 cells were sensitive to the antiproliferative effects, and even high BC concentrations (30 µM) were unable to suppress the growth of the LNCaP line. The same result for BC and the LNCaP cell line was found by Peternac et al. [66]. In our experiment, proliferation decreased in all examined lines, even in LNCaP. Interestingly, in all cases, even low BC doses, which are possible to achieve via dietary supplementation (for example, 3 µM), were able to show significant favorable effects. Moreover, there are also some pieces of evidence that show that BC might have proapoptotic properties (investigated in the colon cancer line HCT-116) [65], which could potentially explain this effect.

The loss of AR in advanced PC leads to resistance to hormonal treatment, leading to the deterioration of the disease [67]. Currently, there are no clinically relevant methods to overcome this loss; only some studies on few cell lines were conducted [65]. From a clinical point of view, AR re-expression could be a desired effect in the treatment of late stages of PC, as it allows regaining control of the disease, thus achieving re-sensitization to hormonal treatment [68].

The results suggest an anti-inflammatory role of BC. The QR-PCR analyses showed that BC downregulates TNF-α mRNA levels in androgen-dependent carcinoma Du145 cells and alleviates the inflammation caused by LP treatment. The Western blot assays indicated a long-term decrease in NF-kB activity, contributing to the anti-inflammatory effect of BC. Carotenoids are known antioxidant factors, and many cellular studies have shown their anti-inflammatory effect, also including a decrease in NF-κB pathway activity [69]. In contrast to these research findings, Chadid et al. found no association between BC serum concentration (at a mean serum BC of 0.41 (0.34–0.48) µM) and intraprostatic inflammation [70].

## 5. Conclusions

Our study has shed some light on the potential of BC in the context of PCa.

We found that BC influenced the viability, migration potential, and connective tissue cleavage capabilities of various PCa cell lines and a healthy prostate model. LP-BCs demonstrated higher antiproliferative capabilities and a more pronounced EMT inhibition compared to BC alone. This suggests that LP-BCs may be a more effective form for PCa prevention or treatment. Furthermore, LPs may modulate lipid metabolism in PCa cells, offering additional benefits.

In conclusion, our findings highlight the potential of BC, particularly in its LP-BC form at physiological concentrations, as a promising direction for research on the prevention and treatment of PCa. However, more studies are needed to fully understand the mechanisms underlying these effects and to evaluate the clinical efficacy of LP-BCs in patients with PCa.

## Figures and Tables

**Table 1:** Main characteristics of the cell lines used in the study.

Cell Line	22Rv1	RWPE	LNCaP	PC-3	Du145
Histological origin of cells	Human prostate carcinoma epithelial cell line derived from a xenograft, CWR22R	Epithelial cells derived from the peripheral zone of histologically normal adult prostate	Human prostate carcinoma cells		
Origin of tissues the cells came from	Xenograft that was serially propagated in mice after castration-induced regression and relapse of the parental, androgen-dependent CWR22 xenograft	Obtained from a healthy, 54-year-old Caucasian man	Obtained from a metastatic lesion in a lymph node of a 50-year-old Caucasian man	Obtained from a bone metastasis in a 62-year-old man	Obtained from a metastasis to the brain of androgen-unresponsive prostate carcinoma in a 69-year-old man
Type of growth	Grown as an adherent culture				
Tumorigenicity in mice	Tumorigenic	Not tumorigenic	Tumorigenic	Tumorigenic	Tumorigenic
Presence of AR and PSA	AR+ PSA+ growth inhibited by androgen withdrawal	AR+ PSA+ AR is upregulated after the exposure to androgens	AR+ PSA+ growth inhibited by androgen withdrawal	AR− PSA− growth independent of androgens	AR− PSA− androgen-independent

Abbreviations: AR—androgen receptor; PSA—prostate-specific antigen.

**Table 2:** Characteristics of primary antibodies used for the Western blot analysis.

Target of Primary Antibody	Host Species	Dilution (at Application)	Vendor
β-actin	mouse	1:12,000	Sigma Aldrich
ZO-1	rabbit	1:1000	Cell Signaling Technology
Snail	rabbit	1:1000	Cell Signaling Technology
Slug	rabbit	1:1000	Cell Signaling Technology
Vimentin	rabbit	1:1000	Cell Signaling Technology
GSK	mouse	1:1000	Cell Signaling Technology
Twist	mouse	1:1000	Sigma Aldrich
ZEB	rabbit	1:1000	Cell Signaling Technology
N-cadherin	mouse	1:1000	Cell Signaling Technology
E-cadherin	mouse	1:1000	Cell Signaling Technology
SREB	rabbit	1:500	AB Clonal
LDL-R	rabbit	1:500	AB Clonal
AR	rabbit	1:1000	Cell Signaling Technology
NF-κB	rabbit	1:500	AB Clonal
LXR	rabbit	1:500	AB Clonal
Akt	mouse	1:500	BD Transduction Laboratories

**Table 3:** Results of the ELISA-LDH cytotoxicity assay. No cytotoxic effect was noted for the evaluated concentrations of BC and/or LPs.

Cell Line	Time of Incubation	Cytotoxicity (% of Dead Cells)							
		1.5 μM BC	3.0 μM BC	5.0 μM BC	7.5 μM BC	15.0 µM BC	30.0 μM BC	LPs	3.0 μM LP-BCs
LNCaP	24 h	0.34	0.44	0.91	1.40	2.50	6.40	0.24	0.45
	48 h	0.39	0.33	1.10	1.90	0.90	5.90	0.47	0.28
PC-3	24 h	0.20	0.30	0.35	0.50	4.50	12.50	0.45	0.34
	48 h	0.25	0.40	0.55	0.80	5.80	12.95	0.45	1.50
Du145	24 h	0.15	0.60	1.50	2.00	6.00	7.55	0.95	2.10
	48 h	0.35	0.95	1.85	1.90	5.39	6.55	1.80	2.90
22Rv1	24 h	0.15	0.85	1.55	7.55	8.00	9.55	0.55	0.65
	48 h	0.65	2.55	3.35	7.85	8.20	8.65	0.75	0.45

Abbreviations: BC—β-carotene, ELISA-LDH—enzyme-linked immunosorbent assay-lactate dehydrogenase; LPs—liposomes; LP-BCs—β-carotene-loaded liposomes.

## Data Availability

The datasets used and/or analyzed during the current study are available from the corresponding author upon reasonable request.

## Supplementary Materials

The following supporting information can be downloaded at: https://www.mdpi.com/article/10.3390/antiox13080902/s1. Supplementary Figure S1: A heatmap presenting the relative gene expression levels measured with the RT-qPCR method with reference to the control samples after 24 h and 48 h. Supplementary Figure S2: Uptake of BC by prostate cells incubated for 48 h. Examples of HPLC determination: A. Standard BC—5 µM; B. BC—0.49 µM; C. BC—2.98 µM; D. LP-BCs—3.5 µM.
