# Supplementary figures and images for: The Influence of β-Carotene and Its Liposomal Form on the Expression of EMT Markers and Androgen-Dependent Pathways in Different Prostate Cell Lines
Source: Antioxidants (Basel). 2024 Jul 25;13(8):902. doi: 10.3390/antiox13080902 (PMC11351549; doi:10.3390/antiox13080902)

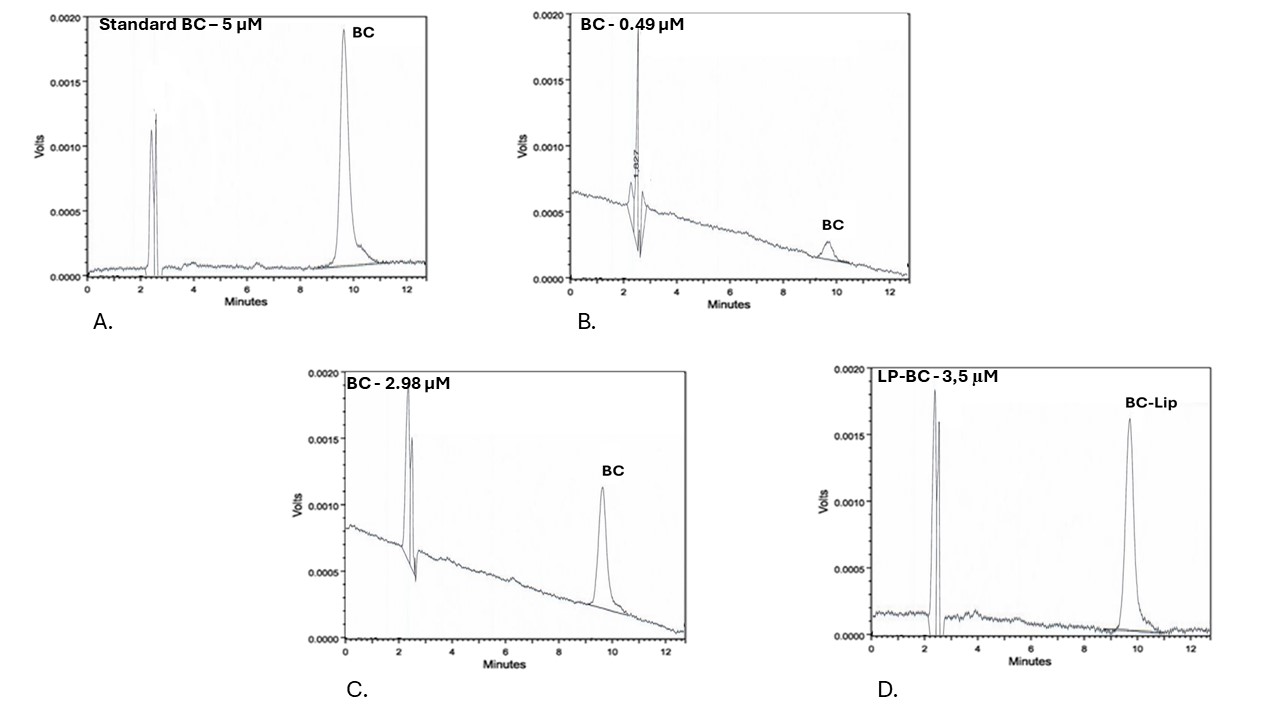

Supplement: Supplementary file 1 [file antioxidants-13-00902-s001.zip › Supplementary Fig/Supplementary Figure. 2. HPLC -BC.jpg]

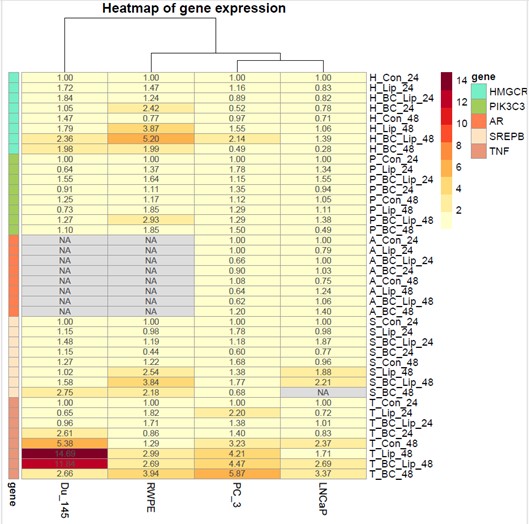

Supplement: Supplementary file 1 [file antioxidants-13-00902-s001.zip › Supplementary Fig/Supplementary Figure 1.jpg]
